# Gastrodin Inhibits Store-Operated Ca^2+^ Entry and Alleviates Cardiac Hypertrophy

**DOI:** 10.3389/fphar.2017.00222

**Published:** 2017-04-25

**Authors:** Changbo Zheng, Chun-Yin Lo, Zhaoyue Meng, Zhichao Li, Mingkui Zhong, Peng Zhang, Jun Lu, Zhaoxiang Yang, Fuman Yan, Yunting Zhang, Yu Huang, Xiaoqiang Yao

**Affiliations:** ^1^Li Ka Shing Institute of Health Sciences and School of Biomedical Sciences, Faculty of Medicine, The Chinese University of Hong KongHong Kong, China; ^2^Shenzhen Research Institute, The Chinese University of Hong KongShenzhen, China; ^3^School of Life Sciences, The Chinese University of Hong KongHong Kong, China; ^4^Institute for Drug Research and Development, Kunming Pharmaceutical CorporationKunming, China

**Keywords:** gastrodin, hypertrophy, cardiomyocyte, SOCE, STIM1, Orai1

## Abstract

Cardiac hypertrophy is a major risk factor for heart failure, which are among the leading causes of human death. Gastrodin is a small molecule that has been used clinically to treat neurological and vascular diseases for many years without safety issues. In the present study, we examined protective effect of gastrodin against cardiac hypertrophy and explored the underlying mechanism. Phenylephrine and angiotensin II were used to induce cardiac hypertrophy in a mouse model and a cultured cardiomyocyte model. Gastrodin was found to alleviate the cardiac hypertrophy in both models. Mechanistically, gastrodin attenuated the store-operated Ca^2+^ entry (SOCE) by reducing the expression of STIM1 and Orai1, two key proteins in SOCE, in animal models as well as in cultured cardiomyocyte model. Furthermore, suppressing SOCE by RO2959, Orai1-siRNAs or STIM1-siRNAs markedly attenuated the phenylephrine-induced hypertrophy in cultured cardiomyocyte model. Together, these results showed that gastrodin inhibited cardiac hypertrophy and it also reduced the SOCE via its action on the expression of STIM1 and Orai1. Furthermore, suppression of SOCE could reduce the phenylephrine-induced cardiomyocyte hypertrophy, suggesting that SOCE-STIM1-Orai1 is located upstream of hypertrophy.

## Introduction

Cardiomyocytes show hypertrophic growth and remodeling in response to prolonged mechanical stress or abnormal neurohumoral activation (Gupta et al., 2007). Cardiac hypertrophy is often maladaptive and contributes to progressive cardiac dysfunction and heart failure (Gupta et al., 2007), which are the leading causes of morbidity and mortality in the world.

Gastrodin (*p*-hydroxymethylphenyl-β-D-glucopyranoside) is the main bioactive ingredient of herbal Chinese medicine *Gastrodia elata B1*. Gastrodin in its purified and synthetic forms has been used clinically to treat neurological and vascular diseases, such as epilepsy, convulsions, migraine, hypertension and others (Hsieh et al., 2001; Zhu et al., 2012). Its mechanism in the nervous and vascular system may be related to *gamma*-aminobutyric acid (GABA), glutamate and calcitonin gene-related peptide (An et al., 2003; Zeng et al., 2006; Luo et al., 2011). A report suggests that gastrodin may attenuate the cardiac hypertrophy induced by aortic banding (Shu et al., 2012), highlighting an interesting possibility of using gastrodin as a potential anti-hypertrophic agent. However, the underlying molecular mechanism for the protective effect of gastrodin against cardiac hypertrophy remains largely unknown.

The literature shows that pathological cardiac hypertrophy may result from the dysregulation of multiple cellular factors and/or signaling pathways, including G protein-coupled receptors, cytosolic Ca^2+^ signaling, and many others (Gupta et al., 2007; Collins et al., 2013; Seo et al., 2014). Store-operated Ca^2+^ entry (SOCE) is an important Ca^2+^ entry pathway, in which depletion of intracellular Ca^2+^ stores stimulates Ca^2+^ entry from the extracellular milieu (Lewis, 2011). The molecular determinants of SOCE include STIM1 and Orai1, in which STIM1 serves as a Ca^2+^ sensor in sarco/endoplasmic reticulum whereas Orai1 functions as the pore-forming subunit for Ca^2+^ permeation. Previous studies from us and others presented the data consistent with the hypothesis that SOCE and its molecular determinants STIM1 and Orai1 can promote hypertrophy in rat neonatal cardiomyocytes, adult cardiomyocytes and embryonic stem-cell derived cardiomyocytes (Hunton et al., 2002; Voelkers et al., 2010; Hulot et al., 2011; Luo et al., 2012; Wang et al., 2015).

In the present study, we investigated the molecular mechanisms underlying the protective effect of gastrodin against cardiac hypertrophy. Our results demonstrated that gastrodin attenuated the phenylephrine-and angiotensin II-induced hypertrophy and also reduced SOCE via its action on the expression levels of Orai1 and STIM1 proteins.

## Materials and Methods

### Animals

All animal experiments were conducted under the authority of a license issued by the Government of the Hong Kong SAR and approval from the Animal Experimentation Ethics Committee, the Chinese University of Hong Kong. Male Sprague-Dawley (S/D) rats (1–2 Days) and male C57BL/6 mice (8 weeks) were provided by Laboratory Animal Services Centre of the Chinese University of Hong Kong.

### Cardiac Hypertrophic Mouse Model

All animals were obtained from the Laboratory of Animal Services Center, the Chinese University of Hong Kong, Hong Kong, China. The animal experiments were conducted in accordance with the Guide for the Care and Use of Laboratory Animals published by the US National Institute of Health. Eight-week-old male C57 mice were sham-operated or subcutaneously infused with phenylephrine at a rate of 70 mg/kg/day for 2 weeks or infused with angiotensin II at a rate of 1.5 mg/kg/day by osmotic mini-pumps (Alza Corp, Alzet model 1002, Cupertino, CA, USA) to establish the hypertrophic model. The control mice were infused with the physiological saline by osmotic mini-pump. When needed, gastrodin was intraperitoneally injected daily at a dose of 50 mg/kg/day starting 1 week before PE.

### Hypertrophic Model of Cultured Cardiomyocytes

Cardiomyocytes were isolated from 1-to 2-day-old neonatal Sprague-Dawley rats using a conventional method as described elsewhere (Wollert et al., 1996). Briefly, rat hearts were dissected and digested by trypsin. The dissociated cells were suspended in Dulbecco’s modified Eagle’s medium [Nutrient Mixture F-12 (DMEM/F12)] with GlutaMAX (GIBCO, Grand Island, NY, USA, 10565018) with 10% horse serum (GIBCO, 16050122), 5% FBS (GIBCO, 11573397), 50 μg/ml gentamicin (GIBCO, 15710072) and cultured in a humidified incubator (95% air with 5% CO_2_) for 60 min to allow selective adhesion of cardiac fibroblasts to culture-ware. Non-adhesive cardiomyocytes in suspension were transferred to another dish and cultured for 24 h in a serum-free MEM with 10 μg/mL transferrin, 10 μg/mL insulin, 1 mg/mL BSA (MEM-TI- BSA), and 0.1 mmol/L BrdU (Deng et al., 2000). The resulting neonatal rat cardiomyocytes (NRCMs) were then treated with 50 μM phenylephrine for 48 h to induce hypertrophy. Up to 85% of cells were α-actinin positive at day 2.

### Cardiac Fibrosis

Cardiac fibrosis was accessed by collagen accumulation as determined using picrosirius red staining. Briefly, heart sections were stained for 1 h with 0.1% (wt/vol) Sirius red (Sigma, St Louis, MO, USA) in a saturated aqueous solution of picric acid (Wako, Osaka, Japan). After staining, the slides were rinsed with two changes of acidified water [0.5% (wt/wt) glacial acetic acid in H_2_O], and then dehydrated in three changes of 100% ethanol. The slides were cleared in xylene, mounted in a resinous medium, and then observed under a light microscope. Sirius red–positive areas were measured using the ImageJ.

### Drugs

When appropriate, gastrodin (100 μmol/L, dissolved in PBS), RO2959 (Glixx Laboratories, Southborough, MA, USA, GLXC-01511) (5 μmol/L, dissolved in DMSO) were added 12 h before phenylephrine (Sigma-Aldrich, St. Louis, MO, USA, P6126) (50 μmol/L, dissolved in PBS), angiotensin II (Tocris, Bristol, UK, 1158) (10 nmol/L, dissolved in water) and endothelin-1 (Tocris, Bristol, UK,1160) (100 nmol/L, dissolved in water) application. The culture media containing different drugs were renewed every 24 h.

### Western Blot

Cellular and tissue proteins were extracted with lysis buffer, which contained 1% (vol/vol) Nonidet P-40, 150 mmol/L NaCl, 20 mmol/L Tris-HCl, pH 8.0, with the addition of Roche protease inhibitor cocktail tablets (Sigma–Aldrich, St. Louis, MO, USA, 04693132001). The protein concentrations were determined with the DC Protein Assay (Bio-Rad, Hercules, CA, USA, 500-0002). The proteins were separated in 12% SDS-PAGE and transferred to PVDF membranes. After blocking with 5% BSA in TBS for 2 h at room temperature, the membranes were incubated with primary antibodies at 4°C overnight. The primary antibody was anti-GAPDH (1:5000, Abcam, Cambridge, UK, ab8245), anti-Orai1 (1:1000, Sigma–Aldrich, St. Louis, MO, USA, O8264), anti-STIM1 (1:1000, Cell Signaling, Danvers, MA, USA 5668S), anti-ANF (1:3000, Abcam, ab14348), or anti-cTnT (1:3000, Abcam, ab8295). Immunodetection was accomplished using horseradish peroxidase-conjugated secondary antibody, followed by analysis via the ECL detection system. The intensity of immunoblot bands was quantified using ImageJ Software.

### Measurement of Cardiomyocyte Surface Area

Cell surface area of NRCMs was measured as described elsewhere (Xu et al., 2014). Briefly, after treatment with different agents, the cells were fixed with 4% paraformaldehyde for 1 h at 4°C, then permeabilized with 0.2% Triton X-100 in PBS for 15 min at room temperature. After blocking with 5% BSA for 2 h, the cells were incubated with anti-α-actinin antibody (abcam, ab18061) overnight at 4°C, followed by incubation with donkey anti-mouse AlexaFluor 555-conjugated secondary antibody (Thermo Fisher Scientific, A-21422) for 2 h at room temperature. The nuclei were counterstained with DAPI (4′,6-diamidino-2-phenylindole, Life Technologies, D1306). The cells were visualized under a FV1000 laser scanning confocal microscope. The areas of 100 randomly selected cells were determined using ImageJ software.

### siRNA Transfection

Neonatal rat cardiomyocytes were seeded at a density of 1.2 × 10^6^/well in a 6-well dish. Twenty four hours after plating, the cells were incubated with 120 nmol/L Orai1-siRNA or 120 nmol/L STIM1-siRNA and 3 μL of RNAiMax (GIBCO, 13778100) in 1 mL Opti-MEM medium (GIBCO, 51985034) for 6 h. One milliliter of serum-free DMEM containing GlutaMAX was then added, followed by an incubation of 24 h. The cells were then subjected to phenylephrine. Small interfering RNAs (Orai1-siRNA, L-081151-02-0005, STIM1-siRNA, L-083718-02-0005, and scrambled-siRNA, D-001810-01-05) were purchased from GE Dharmacon (Lafayette, CO, USA).

### Cytosolic Ca^2+^ Measurement

The cytosolic Ca^2+^ was measured as described elsewhere (Qi et al., 2015). Briefly, NRCMs were loaded with 5 μmol/L Fluo-4/AM (Invitrogen, Waltham, MA, USA) for 40 min in normal Tyrode’s solution that contained in mmol/L, 140 NaCl, 5.4 KCl, 1 MgCl_2_, 2 CaCl_2_, 5.5 glucose and 5 HEPES at pH 7.4. To deplete intracellular Ca^2+^ stores, the cells were treated with 4 μmol/L thapsigargin (TG) in Ca^2+^-free Tyrode’s solution, which contained in mmol/L, 140 NaCl, 5.4 KCl, 1 MgCl_2_, 5.5 glucose, 0.2 ethylene glycol tetraacetic acid (EGTA), 5 HEPES at pH 7.4. SOCE was initiated by addition of 1 mmol/L Ca^2+^ to the bath. The real-time fluorescent images were captured every 10 s and analyzed by MetaFluor imaging software (Molecular Devices, USA).

### Measurement of Vascular Tension

Isometric tension of aortic rings was recorded by Multi Myograph System (Danish Myo Technology A/S, Denmark). Briefly, after euthanasia, the mouse thoracic aorta was quickly dissected out and cut into 2 mm-long rings in ice-cold and oxygenated Krebs buffer, which contained in mmol/L: 119 NaCl, 4.7 KCl, 2.5 CaCl_2_, 1 MgCl_2_, 25 NaHCO_3_, 1.2 KH_2_PO_4_, and 11 D-glucose. The vascular rings were mounted onto two thin stainless steel holders in myograph filled with Krebs solution bubbled with 95% O_2_ and 5% CO_2_ at 37°C. The optimum baseline tension was set at 3 mN. After an equilibration period of 60 min, each endothelium-intact ring was contracted with 60 mmol/L K^+^ at 30-min intervals until two consecutive contractions were similar in amplitude (<10%). Thereafter, the contractile response to phenylephrine (Phe, 1 μmol/L), angiotensin II (Ang-II, 50 nmol/L) or endothelin-1 (ET-1, 50 nmol/L) was measured. The rings were subsequently challenged with acetylcholine to verify the integrity of endothelium.

### Statistical Analysis

All values are expressed as the mean ± SEM (n), where n corresponds to the number of independent experiments. The significant differences were determined using paired or unpaired Student’s *t*-test for comparison of two groups, or one-way ANOVA followed by the Newman–Keuls test for comparison of multiple groups, or two-way ANOVA followed by Bonferroni post-test for comparison of multiple groups in **Figure 3**, using GraphPad Prism software (GraphPad Software, Inc., San Diego, CA, USA). The differences were considered statistically significant at *P* < 0.05.

## Results

### Gastrodin Protected against Phenylephrine- or Angiotensin II-Induced Cardiac Hypertrophy *In Vivo* and *In Vitro*

**FIGURE 1:**
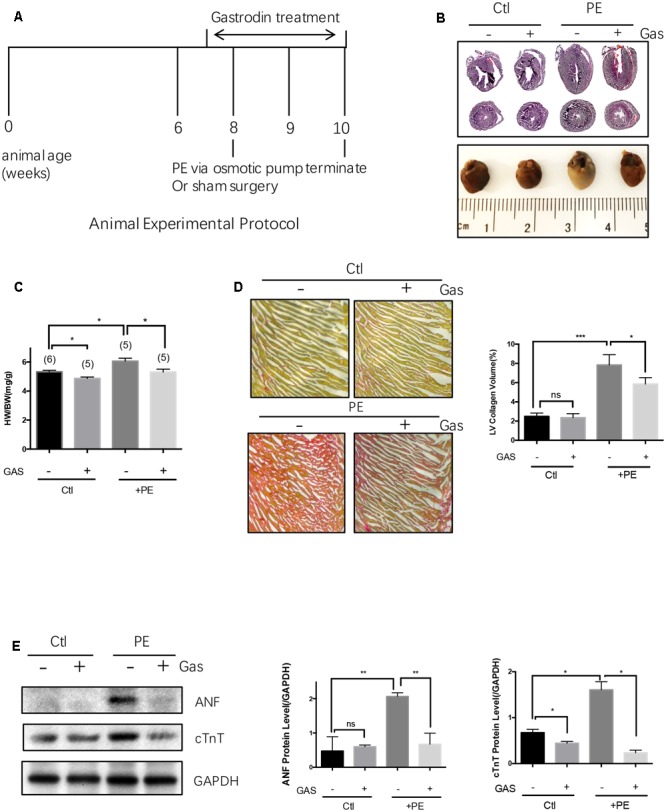
**Gastrodin inhibited the phenylephrine (PE)-induced cardiac hypertrophy and fibrosis *in vivo.* Mouse cardiac hypertrophic model. (A)** Shows the schematic experimental protocol. The mice were infused with PE at a dose of 70 mg/kg/day for 2 weeks. The gastrodin (GAS, 50 mg/kg/day) injection started 1 week before PE. The control group (Ctl) did not undergo PE treatment. Hypertrophy was assessed by measuring heart size (**B**, representative from 6 pairs of mice) and the ratio of heart weight (HW)/total body weight (BW) (**C**, *n* = 5–6). **(D)** Shows the change in collagen content in mouse hearts with representative images and data summary (*n* = 5). Sections are stained with picrosirius red and collagen fibers appear red. **(E)** Shows the changes in ANF and cTnT protein expression with representative images and data summary (*n* = 5). The data are expressed as the mean ± SEM (*n* = 5–6 repeats). ^∗^*p* < 0.05; ^∗∗^*p* < 0.01; ^∗∗∗^*p* < 0.001. ns, not significant.

Continuous infusion of commonly used hypertrophic inducer phenylephrine (70 mg/kg/day) or angiotensin II (1.5 mg/kg/day) to C57 mice via an implantable Alzet osmotic pump for 2 weeks resulted in cardiac hypertrophy, as indicated by an enlargement in heart size and an increased ratio of heart weight/total body weight (**Figures 1A–C** and **Supplementary Figures S1A,B**). Phenylephrine infusion also caused cardiac fibrosis as indicated by abnormal accumulation of collagen fibers with collagen volume fraction increased from 2.5 to 7.8% (**Figure 1D**). Pretreatment with gastrodin (50 mg/kg/day starting 1 week before the phenylephrine or angiotensin II application) reduced the phenylephrine- or angiotensin II-induced heart size enlargement (**Figures 1A–C** and **Supplementary Figures S1A,B**) as well as phenylephrine-induced cardiac fibrosis (**Figure 1D**). In the absence of hypertrophic stimuli, the effect of gastrodin on hear size varied, showing inhibition in **Figure 1C** but no effect in **Supplementary Figure S1B**. After combining two data sets from **Figure 1C** and **Supplementary Figure S1B**, gastrodin had no overall effect on hear size (5.0 ± 0.1 mgHW/gBW, *n* = 11 untreated animals vs. 4.8 ± 0.1 mgHW/gBW, *n* = 10 gastrodin-treated animals; *P* = 0.15 by Student’s *t*-test).

Cardiac hypertrophy *in vivo* was also analyzed by two common hypertrophic markers atrial natriuretic factor (ANF) and cardiac troponin T (cTnT) at protein levels (**Figure 1E**). Phenylephrine infusion (70 mg/kg/day) to C57 mice increased the expression levels of these two hypertrophic markers in heart tissue, the effect of which was markedly attenuated by gastrodin (50 mg/kg/day) (**Figure 1E**). However, in the absence of hypertrophic stimuli, the effect of gastrodin on the expression of ANF and cTnT varied, showing small inhibition on cTnT expression but no effect on ANF expression (**Figure 1E**).

**FIGURE 2:**
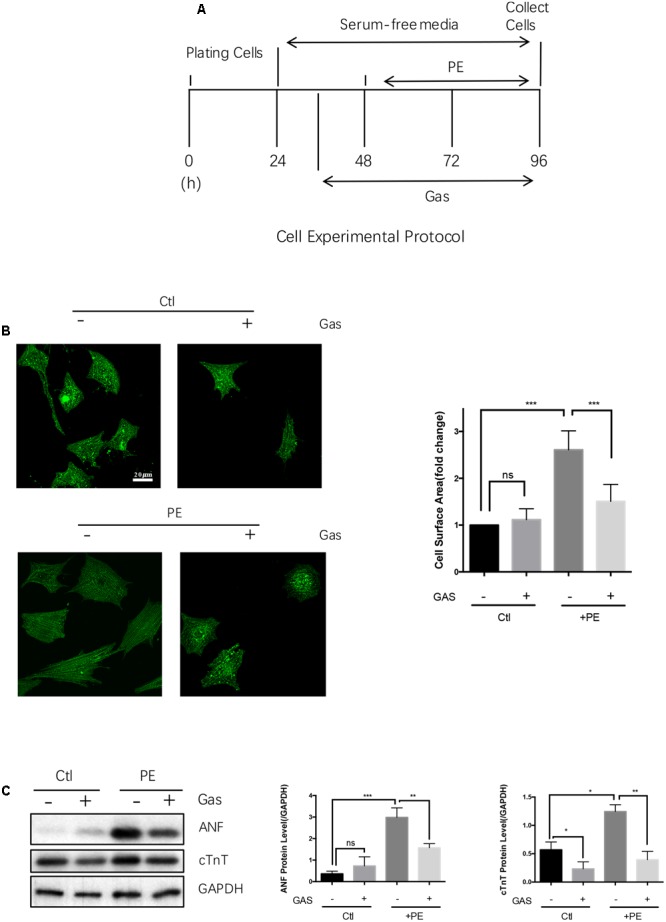
**Gastrodin inhibited the PE-induced cardiac hypertrophy in cultured rat neonatal cardiomyocytes.** Cultured NRCMs. **(A)** Shows the schematic experimental protocol. NRCMs were treated with 50 μmol/L PE for 48 h to induce hypertrophy. GAS at 100 μmol/L was applied 12 h before PE. **(B)** Shows the changes in cell surface area with representative images (left) and data summary (right). The cells were stained with anti-α-actinin antibody (green). **(C)** Shows the changes in ANF and cTnT protein expression with representative images (left) and data summary (right). The data are expressed as the mean ± SEM [*n* = 5 repeats, >50 cells per repeat in **(B)**; *n* = 5 repeats in **(A–C)**]. ^∗^*p* < 0.05; ^∗∗^*p* < 0.01; ^∗∗∗^*p* < 0.001. ns, not significant.

Phenylephrine treatment (50 μmol/L, 48 h) also induced hypertrophic growth in the primary cultured NRCMs, as indicated by an increased cell surface area (**Figures 2A,B**) and enhanced expressions of ANF and cTnT at protein level (**Figure 2C**). Pretreatment with gastrodin (100 μmol/L, applied 12 h before phenylephrine) markedly reduced the phenylephrine-induced hypertrophy in NRCMs (**Figures 2A–C**). Similarly, angiotensin II (10 nmol/L, 48 h) and endothelin-1 (100 nmol/L, 48 h) enhanced the expression of a hypertrophic marker ANF in the primary cultured NRCMs, the effect of which was attenuated by gastrodin (100 μmol/L) (**Supplementary Figure S1C**). However, in the absence of hypertrophic stimuli, gastrodin had no effect on the size of NRCMs (**Figure 2B**). In the absence of hypertrophic stimuli, the effect gastrodin on the expression of ANF and cTnT in NRCMs varied, showing small inhibition on cTnT expression but no effect on ANF expression (**Figure 2C**).

In another control, we found that gastrodin had no direct inhibitory effect on α-adrenergic receptors, angiotensin receptors or endothelin-1 receptors, because phenylephrine-, angiogensin II- or endothelin 1-induced contraction of mouse aortas was not altered by 100 μmol/L gastrodin (**Supplementary Figure S2**).

### Gastrodin Attenuated the Phenylephrine-Induced Cardiomyocyte Hypertrophy in a Dose-Dependent Manner

**FIGURE 3:**
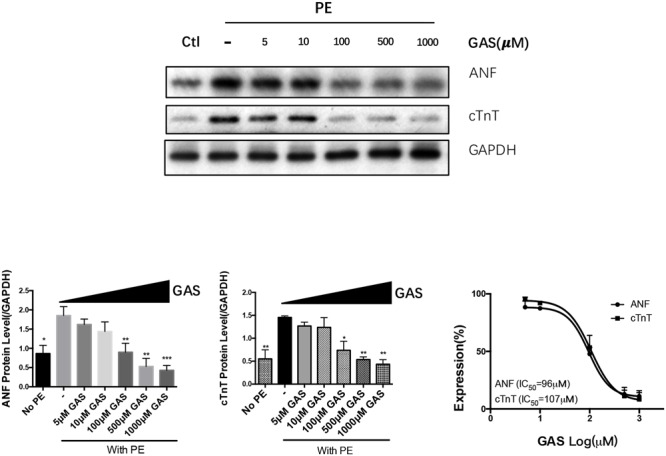
**Dose-dependent inhibition of gastrodin on phenylephrine-induced hypertrophy of NRCMs.** NRCMs were treated with 50 μmol/L PE for 48 h to induce hypertrophy. GAS (0–1000 μmol/L) was applied 12 h before PE. The control group (no PE) did not undergo PE treatment. Effect of gastrodin on the expression of ANF and cTnT was demonstrated. Shown were representative immunoblot images **(upper panel)**, data summary **(lower panels)** and determination of IC_50_ values **(lower right)**. The data are expressed as the mean ± SEM (*n* = 5). ^∗^*p* < 0.05, ^∗∗^*p* < 0.01, ^∗∗∗^*p* < 0.001.

We examined the effect of different dose of gastrodin (0–1000 μmol/L) on the phenylephrine-induced hypertrophy in NRCMs. The results show that gastrodin caused a dose-dependent inhibition on ANF and cTnT expression with IC_50_ of 96 μmol/L and 107 μmol/L, respectively (**Figure 3**).

### SOCE Is Essential for Phenylephrine-Induced Cardiomyocyte Hypertrophy

**FIGURE 4:**
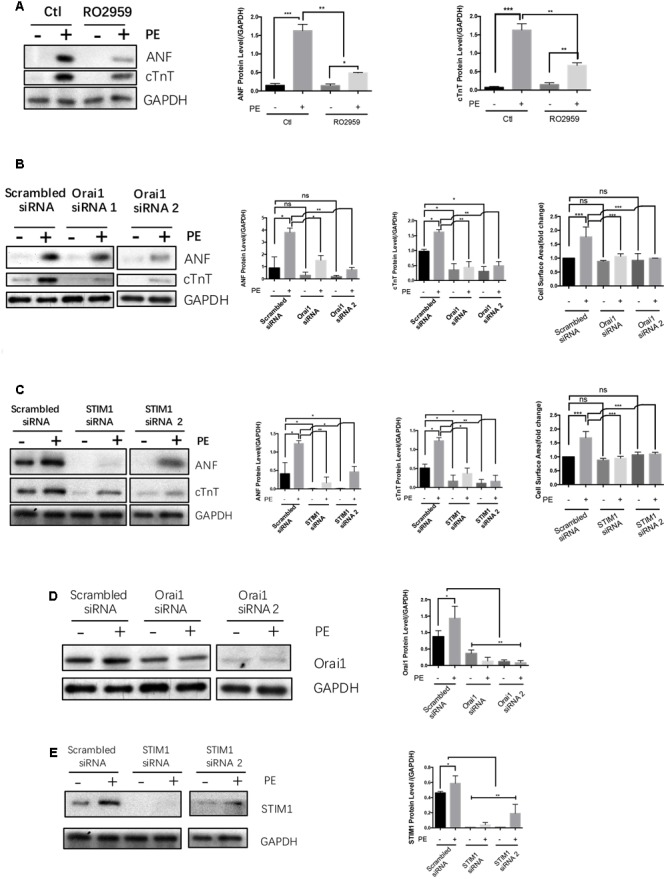
**SOCE/STIM1/Orai1 were linked to hypertrophy in NRCMs.** NRCMs were treated with 5 μmol/L RO2959, or transfected with Orai1-siRNA, STIM1-siRNA or Scrambled-siRNA, followed by 50 μmol/L PE for 48 h to induce hypertrophy. Negative control group (–) did not undergo PE treatment. **(A)** Effects of RO2959 on PE-induced cardiomyocyte hypertrophy. **(B,C)** Effects of Orai1-siRNAs and STIM1-siRNAs on PE-induced cardiomyocyte hypertrophy. The hypertrophy was assessed by protein expression of ANF and cTnT **(A–C)** as well as cell surface area **(B,C)**. **(D,E)** Effectiveness of Orai1-siRNAs and STIM1-siRNAs in suppressing the protein expression of their respective targets. Shown were representative Western blot images (left) and data summary (right). The data are expressed as the mean ± SEM (*n* = 5). ^∗^*p* < 0.05; ^∗∗^*p* < 0.01; ^∗∗∗^*p* < 0.001. ns, not significant.

Previous reports suggest that SOCE is an important immediate signal that contribute to agonist-induced cardiac hypertrophy (Hunton et al., 2002; Voelkers et al., 2010; Hulot et al., 2011; Luo et al., 2012; Wang et al., 2015). This was confirmed in the present study. Treatment of NRCMs with a selective Orai1 inhibitor RO2959 (Chen et al., 2013) at 5 μmol/L markedly reduced the phenylephrine-induced hypertrophy in NRCMs, as indicated by reduced protein levels of cTnT and ANF (**Figure 4A**). Furthermore, compared to scrambled-siRNA control, two Orai1-siRNAs and two STIM1-siRNAs all reduced the phenylephrine-induced hypertrophy as indicated by reduced protein levels of cTnT and ANF and reduced cell surface area (**Figures 4B,C**). For control, we verified that Orai1-siRNAs and STIM1-siRNAs could effectively suppress the protein expression of Orai1 and STIM1, respectively (**Figures 4D,E**). These data demonstrated a critical role of SOCE in promoting cardiomyocyte hypertrophy.

In the absence of hypertrophic stimuli, STIM1-siRNA and Orai1-siRNA had no effect on the size of NRCMs (**Figures 4B,C**, left panels). In the absence of hypertrophic stimuli, the effect of STIM1-siRNA and Orai1-siRNA on the expression of ANF and cTnT in NRCMs varied from small inhibition to no effect (**Figures 4B,C**).

### Chronic Gastrodin Treatment Suppressed the Expression of STIM1/Orai1 Proteins and Their Associated SOCE, While Acute Gastrodin Treatment Had No Effect on SOCE

**FIGURE 5:**
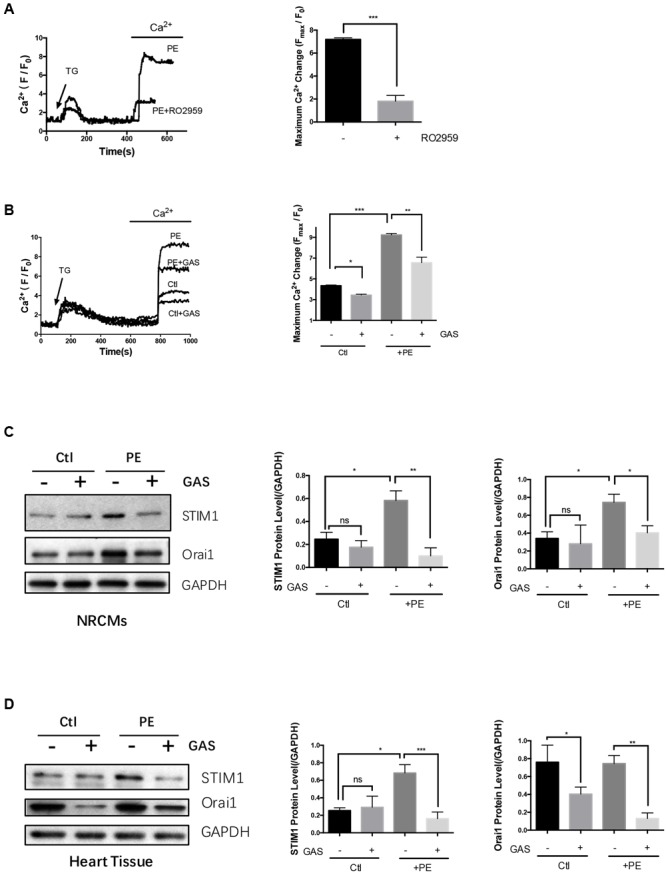
**Chronic gastrodin treatment inhibited SOCE and reduced the expression of STIM1 and Orai1.** NRCMs were treated with 50 μmol/L PE for 48 h to induce hypertrophy. **(A)** Acute effect of RO2959 on SOCE in phenylephrine-treated hypertrophic cardiomyocytes. The cells were treated with 4 μmol/L thapsigargin (TG) in a Ca^2+^-free bath, which induced the first Ca^2+^ rise, followed by adding back 1 mmol/L Ca^2+^ to the bath, which induced the second Ca^2+^ rise. The second Ca^2+^ rise is due to SOCE. RO2959 at 5 μmol/L was applied 5 min before TG. The representative traces (left) and data summary (right) of Ca^2+^ responses are shown. Only SOCE is plotted in the summary chart. **(B)** Effect of chronic gastrodin treatment on SOCE. GAS at 100 μmol/L was applied 12 h before PE. The control group (Ctl) did not undergo PE treatment. **(C,D)** Effect of gastrodin on the protein expression of STIM1 and Orai1 in NRCMs *in vitro* **(C)** and heart tissue *in vivo* **(D)**. The representative images (left) and data summary of immunoblots (right) are shown. GAPDH was used as the house-keeping control gene. The data are expressed as the mean ± SEM [*n* = 5 repeats, >15 cells per repeat in **(A,B)**; *n* = 5 in **(C,D)**]. ^∗^*p* < 0.05; ^∗∗^*p* < 0.01; ^∗∗∗^*p* < 0.001. ns, not significant.

Neonatal rat cardiomyocytes were firstly treated with 4 μmol/L TG in a Ca^2+^-free bath to deplete intracellular Ca^2+^ stores. One mmol/L Ca^2+^ was then added back to induce a cytosolic Ca^2+^ rise, which was taken as SOCE. As expected, 5 μmol/L RO2959 substantially inhibited the SOCE in phenylephrine-treated (50 μmol/L, 48 h) hypertrophic cardiomyocytes by 74% (**Figure 5A**), confirming that Orai1 is the main mediator of SOCE in these cells.

We next explored the possible action of gastrodin on the SOCE. The magnitude of SOCE was larger in the phenylephrine- treated (50 μmol/L, 48 h) hypertrophic cardiomyocytes than in non-treated control cardiomyocytes (**Figure 5B**). In agreement, immunoblots show that STIM1 and Orai1 protein expression was higher in phenylephrine-treated hypertrophic cardiomyocytes than in non-treated cells (**Figure 5C**). Importantly, chronic treatment of NRCMs with gastrodin (100 μmol/L, applied 12 h before phenylephrine) markedly reduced the SOCE (**Figure 5B**) and suppressed the expression of STIM1 and Orai1 (**Figure 5C**) in these hypertrophic cardiomyocytes.

The effect of gastrodin on STIM1 and Orai1 expression was also studied in the mouse model of hypertrophy. Continuous infusion phenylephrine (70 mg/kg/day) via an implantable Alzet osmotic pump for 2 weeks also stimulated the expression of STIM1 proteins but not Orai1. Importantly, gastrodin treatment (50 mg/kg/day starting 1 week before the phenylephrine application) also markedly reduced the expression levels of STIM1 and Orai1 proteins (**Figure 5D**). Taken together, these data indicate that gastrodin acts to suppress the expression of STIM1 and Orai1 proteins in both *in vivo* and *in vitro* models of cardiac hypertrophy.

**FIGURE 6:**
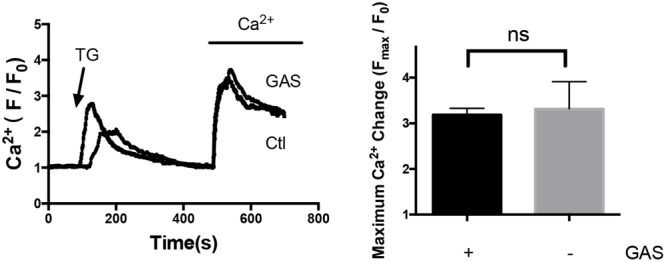
**Lack of acute gastrodin action on SOCE in phenylephrine-treated hypertrophic cardiomyocytes.** Shown are representative immunoblot images **(left)** and data summary **(right)**. The phenylephrine-treated (50 μmol/L, 48 h) hypertrophic cardiomyocytes were first challenged with 4 μmol/L TG in a Ca^2+^-free bath, which induced the first Ca^2+^ rise, followed by adding back 1 mmol/L Ca^2+^ to the bath, which induced the second Ca^2+^ rise. The second Ca^2+^ rise is due to SOCE. Only SOCE is plotted in the summary chart. When appropriate, 100 μmol/L gastrodin was applied 5 min before TG. The data are expressed as the mean ± SEM (*n* = 5 repeats, >15 cells per repeat). ns, not significant.

On the other hand, acute treatment with 100 μmol/L gastrodin for 5 min had no inhibitory effect on SOCE in hypertrophic cardiomyocytes (**Figure 6**). Therefore, the inhibitory effect of gastrodin on SOCE was due to the gastrodin action on the expression level of STIM1 and Orai1 proteins rather than due to its direct action on ion channel activity itself.

## Discussion

The main findings of the present study were as follows: (a) Gastrodin alleviated the phenylephrine-and angiotensin II-induced cardiac hypertrophy in a mouse animal model and a cultured cardiomyocyte model. (b) The inhibitory effect of gastrodin was dose-dependent with IC_50_ of ∼100 μmol/L in the cultured cardiomyocyte model. (c) Suppression of SOCE by an Orai1 inhibitor RO2959, Orai1-siRNAs, or STIM1-siRNAs all attenuated the phenylephrine-induced hypertrophy, demonstrating a critical role of SOCE, STIM1 and Orai1 in promoting cardiac hypertrophy. (d) Phenylephrine treatment increased the magnitude of SOCE and stimulated the expression of STIM1 and Orai1 proteins in the cultured cardiomyocyte model. In both phenylephrine-induced hypertrophic models (animal model and cultured cardiomyocytes), gastrodin treatment substantially inhibited the expressional levels of STIM1 and Orai1 proteins. Taken together, this study demonstrated that gastrodin attenuated the cardiac hypertrophy and suppressed the SOCE via its action on Orai1 and STIM1 expression. Furthermore, suppression of SOCE could reduce the phenylephrine-induced cardiac hypertrophy, suggesting that SOCE is an upstream signaling of cardiac hypertrophy.

Pathological cardiac hypertrophy could either result from pressure overload or due to abnormal activation of neurohormone system including sympathetic nervous system, renin-angiotensin system and endothelins (Jackson et al., 2000; Gupta et al., 2007). Angiotensin II, endothelin-1 and α-adrenergic receptor agonists induce cardiac hypertrophy through G-protein coupled receptors, especially Gαq (Gupta et al., 2007). In addition, evidence shows that the pressure overload-induced cardiac hypertrophy is at least partly mediated through G-protein coupled receptors (Gupta et al., 2007). One previous study suggested that gastrodin attenuated the cardiac hypertrophy induced by pressure overload (Shu et al., 2012). In the present study, we used neurohormonal hypertrophic agents phenylephrine and angiotensin-II to induce cardiac hypertrophy and found that 50 mg/kg/day gastrodin can reduce the cardiac hypertrophy induced by these agents. The anti-hypertrophic effect of gastrodin was demonstrated in a mouse animal model as well as a cultured cardiomyocyte model based on multiple hypertrophic indexes, including heart size, ratio of heart weight/body weight, cardiomyocyte surface area and the expression levels of hypertrophic markers ANF and cTnT. Together, these results raise an intriguing possibility of using gastrodin as a new anti-hypertrophic therapeutic agent. Gastrodin is a small molecule that has been used clinically to treat neurological and vascular diseases for many years without safety issue (Huang, 1985; Hsieh et al., 2001; Zhu et al., 2012). Its clinical information regarding safety dosage and tolerance for gastrodin is already well documented (Huang, 1985). The effective dosage we demonstrated for anti-hypertrophic action, which was 50 mg/kg/day in mice, equivalent to 4 mg/kg/day in human based on the conversion factor of 12.3 (Nair and Jacob, 2016), is within the clinical dosage for neurological disease treatment (200–600 mg/day per adult, or 3.3 – 10 mg/kg/day based on body weight of 60 kg) (Huang, 1985; Nair and Jacob, 2016). In cultured cardiomyocyte model, we used 100 μM gastrodin, which is also near the range of the plasma gastrodin concentration of mice injected with 200 mg/kg gastrodin (with the peak plasma concentration of 500 μM gradually declined to 150 μM 3 h after the injection) (Cai et al., 2014). This should greatly enhance the potential of using gastrodin as a future anti-hypertrophic agent.

Cardiac hypertrophy is traditionally treated with compounds that target cell surface receptors such as β-adrenergic receptors and angiotensin receptors (McKinsey and Kass, 2007; Schiattarella and Hill, 2015). However, their effectiveness seems limited (McKinsey and Kass, 2007). Thus, a more recent trend in anti-hypertrophic therapy focuses on small molecules that act inside the cardiomyocytes targeting crucial signaling cascades that alter cardiomyocyte remodeling, including the inhibitors for histone deacetylase (Schiattarella and Hill, 2015) and G protein-coupled receptor kinase (Traynham et al., 2016). SOCE is a major Ca^2+^ signaling pathway that plays a key role in promoting cardiac hypertrophy (Hunton et al., 2002; Voelkers et al., 2010; Hulot et al., 2011; Luo et al., 2012; Wang et al., 2015). Pro-hypertrophic role of SOCE has been demonstrated both in the pressure overload-induced hypertrophic models (Hulot et al., 2011; Luo et al., 2012) and the neurohormonal agent-induced hypertrophic models (Hunton et al., 2002; Ohba et al., 2009; Voelkers et al., 2010; Wang et al., 2015). Therefore, SOCE and its molecular determinants STIM1 and Orai1 offer attractive targets for anti-hypertrophic therapy. In the present study, we clearly showed that gastrodin could suppress the expressional level of STIM1 and Orai1 proteins, resulting in a reduced SOCE in cardiomyocytes. Furthermore, suppressing SOCE could reduce the phenylephrine-induced cardiomyocyte hypertrophy. Based on these results, it is reasonable to speculate that gastrodin may act through SOCE to exert its anti-hypertrophic effect. Therefore, gastrodin may represent a novel anti-hypertrophic agent that targets intracellular Ca^2+^ handling proteins.

One concern is whether gastrodin could reduce the heart size in the absence of hypertrophic stimuli, which is detrimental. If we combine two sets of data from **Figure 1C** and **Supplementary Figure S1B**, 50 mg/kg/day gastrodin had no statistically significant effect on the heart size of mice in the absence of hypertrophic stimuli (5.0 ± 0.1 mg/g, *n* = 11 untreated animals vs. 4.8 ± 0.1 mg/g, *n* = 10 gastrodin-treated animals; *P* = 0.15 by Student’s *t*-test). This notion was supported by the results from cultured cardiomyocytes, in which gastrodin also had no effect on the cell size of NRCMs in the absence of hypertrophic stimuli (**Figure 2B**). However, caution still need to be taken on the issue, because, in some experiments, gastrodin indeed reduced the cell size (**Figure 1C**) or suppress the expression of cTnT (**Figures 1E, 2C**). Further experiments with larger experimental samples may be needed to clarify the issue. Another related caution is that complete inhibition of SOCE should be detrimental, because SOCE plays important roles in diverse physiological processes in many cell types including cardiomyocytes (Collins et al., 2013; Lewis, 2011). Therefore, for its anti-hypertrophic usage, gastrodin dosage should not be too high.

The main focus of the present study is about gastrodin effect on SOCE and its molecular determinants STIM1-Orai1. Another class of Ca^2+^-permeable channels, TRPC channels, may also be coupled to STIM1, thus contributes to SOCE in some cell types (Collins et al., 2013). However, previous studies suggested that the SOCE in cardiomyocytes is primarily mediated by STIM1-Orai1 rather than STIM1-TRPC channels (Zhu-Mauldin et al., 2012; Collins et al., 2013). In our study, RO2959 inhibited SOCE by 74%, confirming the primary role of STIM1-Orai1 rather than STIM1-TRPC in the SOCE of cardiomyocytes (**Figure 5A**). Therefore, we did not further explore the involvement of TRPC channels in the present study. Nevertheless, pathological cardiac hypertrophy is a complex process that involves multiple cellular factors and signaling pathways (Gupta et al., 2007; Collins et al., 2013; Seo et al., 2014). Although our present results implicate that gastrodin may act through STIM1-Orai1 expression to inhibit cardiac hypertrophy, we cannot exclude the possibility that gastrodin could also act on other hypertrophy-related pathways/factors such as TRPC channels. Further study is needed to further explore other gastrodin targets if any.

## Conclusion

The present study suggested a mechanistic scheme for the protective action of gastrodin against cardiac hypertrophy. We demonstrated that gastrodin attenuated the cardiac hypertrophy and suppressed the SOCE via its action on Orai1 and STIM1 expression. Furthermore, we found that suppressing SOCE could reduce the phenylephrine-induced cardiac hypertrophy. Based on these, we further hypothesize that gastrodin may act through SOCE/STIM1/Orai1 to exert its anti-hypertrophic effect.

## Author Contributions

XY, CZ, and YH conceived and designed the experiments; C-YL, ZM, MZ, PZ, JL, FY, and YZ performed the experiments; CZ, MZ, and JL analyzed the data; ZL and ZY contributed reagents/materials; and CZ and XY wrote the paper.

## Conflict of Interest Statement

ZY is a full-time employee of Kunming Pharmaceutical Group Limited. The other authors declare that the research was conducted in the absence of any commercial or financial relationships that could be construed as a potential conflict of interest.

## Supplementary Material

FIGURE S1 **Gastrodin inhibited the angiotensin II (Ang-II)-induced cardiac hypertrophy *in vivo* and cultured cardiomyocytes *in vitro*. (A,B)**: Mouse cardiac hypertrophic model. The mice were infused with Ang-II at a dose of 1.5 mg/kg/day for 2 weeks. The gastrodin (GAS, 50 mg/kg/day) injection started 1 week before PE. The control group (Ctl) did not undergo PE treatment. Hypertrophy was assessed by measuring heart size (**A**, representative from 6 pairs of mice) and the ratio of heart weight (HW)/total body weight (BW) (**B**, *n* = 5–6). The data are expressed as the mean ± SEM (^∗^*P* < 0.05). **(C)** Cultured NRCMs. NRCMs were treated with 10 nmol/L AngII or 100 nmol/L ET-1 for 48 h to induce hypertrophy. GAS at 100 μmol/L was applied 12 h before PE. **(C)** Shows the changes in ANF and cTnT protein expression with representative images and data summary. The data are expressed as the mean ± SEM (*n* = 5–6 repeats) ^∗^*p* < 0.05; ^∗∗^*p* < 0.01. ns, not significant.

FIGURE S2 **Lack of gastrodin effect on the contractile responses of mouse aortas to PE, Ang-II and endothelin-1.** Shown are representative traces (left) and data summary (right). If needed, the aortas were pretreated with 100 μmol/L gastrodin for 10 min before application of **(A)** phenylephrine (PE, 1 μmol/L), **(B)** angiotensin II (Ang-II, 50 nmol/L) or **(C)** endothelin-1 (ET-1, 50 nmol/L). The magnitude of contraction was normalized to the percentage of contraction in response to 60 mmol K^+^. The data are expressed as the mean ± SEM (*n* = 5–6 repeats). ns, not significant.
