# Supplementary figures and images for: Gastrodin Inhibits Store-Operated Ca2+ Entry and Alleviates Cardiac Hypertrophy
Source: Front Pharmacol. 2017 Apr 25;8:222. doi: 10.3389/fphar.2017.00222 (PMC5404510; doi:10.3389/fphar.2017.00222)

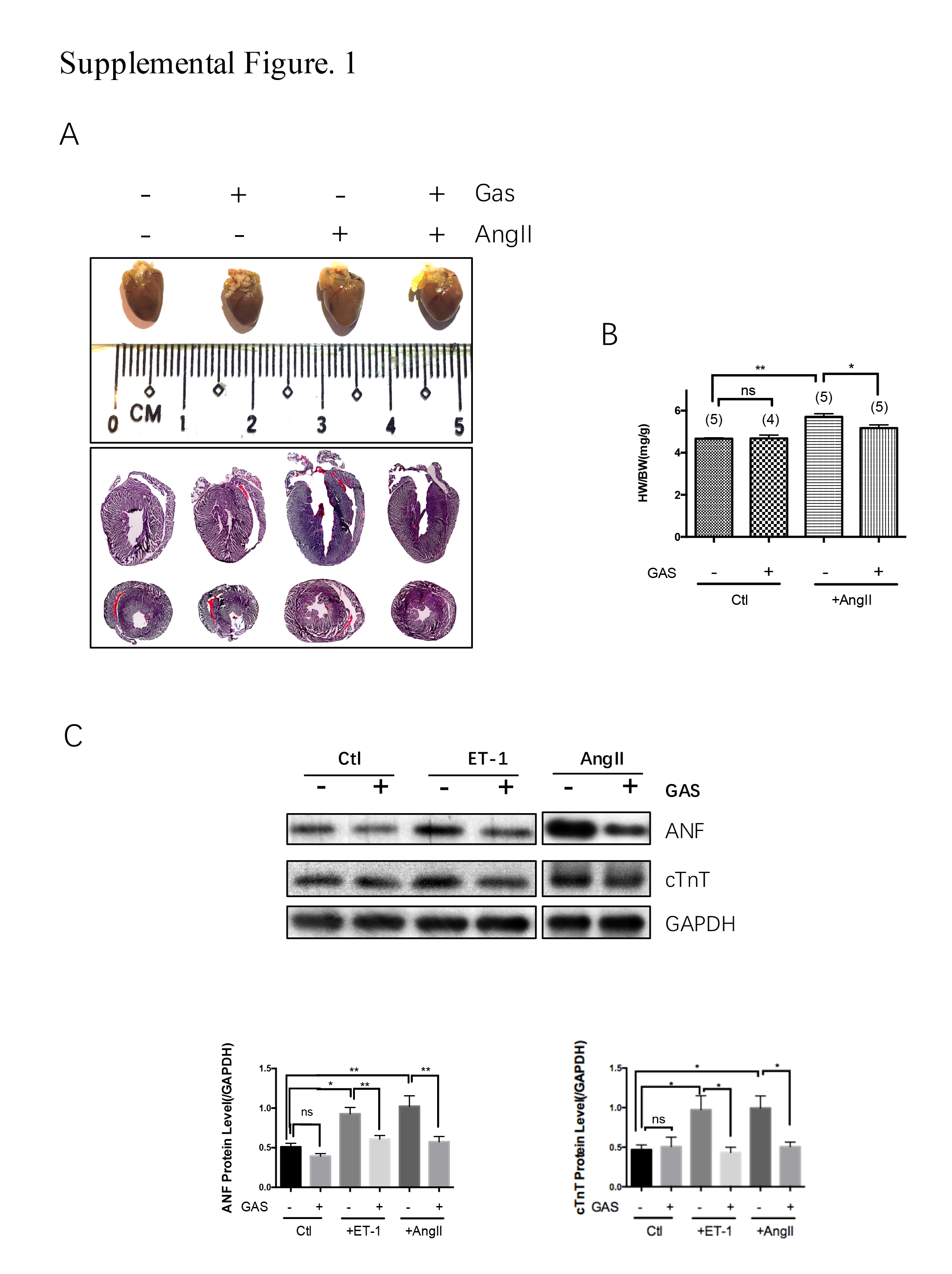

Supplement: FIGURE S1 — Gastrodin inhibited the angiotensin II (Ang-II)-induced cardiac hypertrophy in vivo and cultured cardiomyocytes in vitro. (A,B): Mouse cardiac hypertrophic model. The mice were infused with Ang-II at a dose of 1.5 mg/kg/day for 2 weeks. The gastrodin (GAS, 50 mg/kg/day) injection started 1 week before PE. The control group (Ctl) did not undergo PE treatment. Hypertrophy was assessed by measuring heart size (A, representative from 6 pairs of mice) and the ratio of heart weight (HW)/total body weight (BW) (B, n = 5–6). The data are expressed as the mean ± SEM (∗P < 0.05). (C) Cultured NRCMs. NRCMs were treated with 10 nmol/L AngII or 100 nmol/L ET-1 for 48 h to induce hypertrophy. GAS at 100 μmol/L was applied 12 h before PE. (C) Shows the changes in ANF and cTnT protein expression with representative images and data summary. The data are expressed as the mean ± SEM (n = 5–6 repeats) ∗p < 0.05; ∗∗p < 0.01. ns, not significant. [file Image_1.tif]

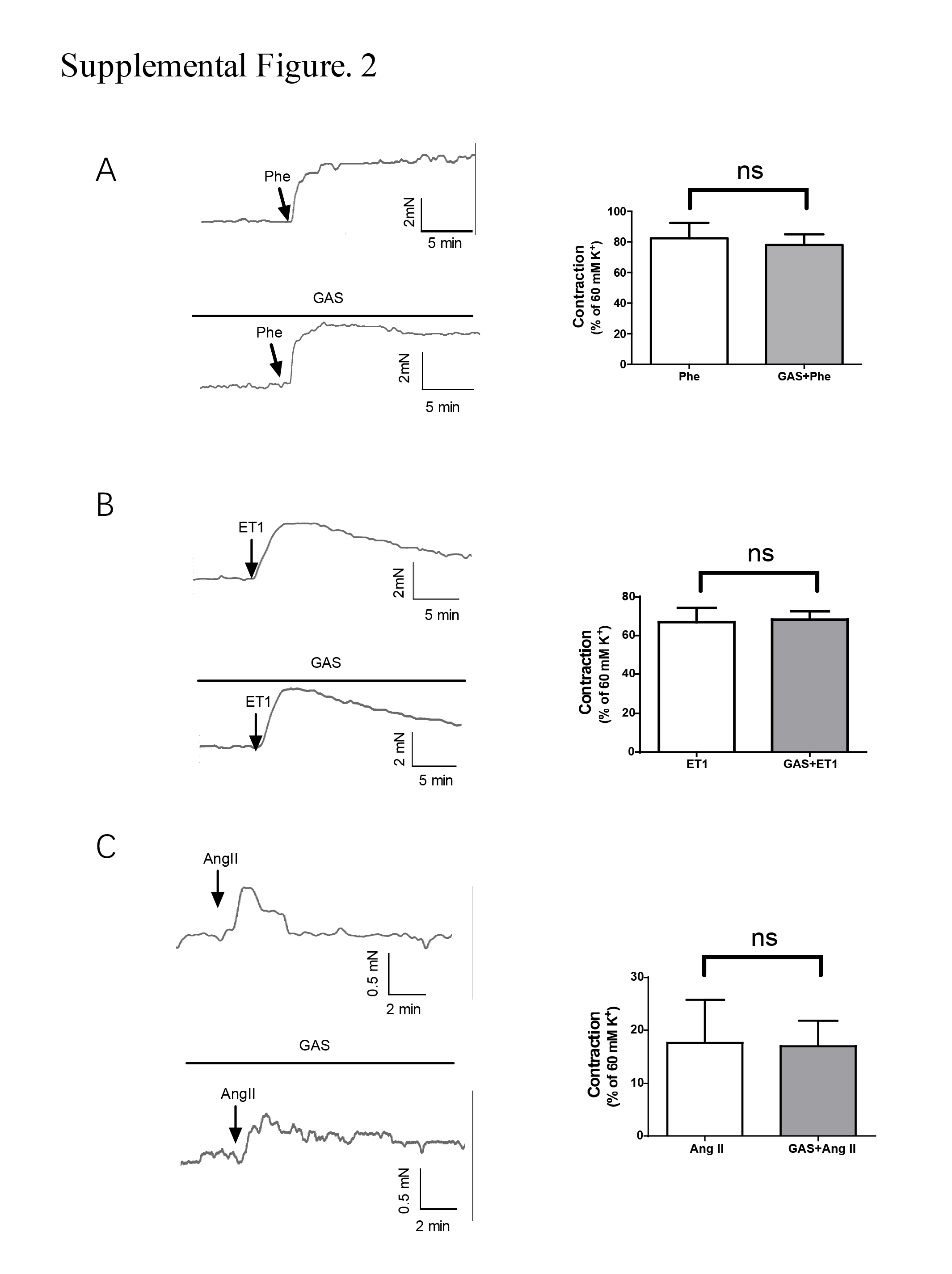

Supplement: FIGURE S2 — Lack of gastrodin effect on the contractile responses of mouse aortas to PE, Ang-II and endothelin-1. Shown are representative traces (left) and data summary (right). If needed, the aortas were pretreated with 100 μmol/L gastrodin for 10 min before application of (A) phenylephrine (PE, 1 μmol/L), (B) angiotensin II (Ang-II, 50 nmol/L) or (C) endothelin-1 (ET-1, 50 nmol/L). The magnitude of contraction was normalized to the percentage of contraction in response to 60 mmol K+. The data are expressed as the mean ± SEM (n = 5–6 repeats). ns, not significant. [file Image_2.TIF]
